# Potential Protection of Pre-Existent Antibodies to Human Coronavirus 229E on COVID-19 Severity

**DOI:** 10.3390/ijerph18179058

**Published:** 2021-08-27

**Authors:** Oscar Guzmán-Martínez, Kathia Guardado, Miguel Varela-Cardoso, Alejandro Trujillo-Rivera, Iván Gómez-Ñañez, María Cristina Ortiz-León, Rafaela Espinosa, Celso Ramos, Julio Isael Pérez-Carreón, Delia Vanesa López-Guerrero, Clara Luz Sampieri, Adrián Baruch Alanís-García, Fausto Rojas-Durán, Roberto Zenteno-Cuevas, Michelle Gutiérrez, Hilda Montero

**Affiliations:** 1 Instituto de Salud Pública, Universidad Veracruzana, Xalapa 91190, Mexico; oscarguzmanmtz@yahoo.com (O.G.-M.); kathia.aguardado@gmail.com (K.G.); qcgomezyanez@outlook.com (I.G.-Ñ.); cortiz@uv.mx (M.C.O.-L.); csampieri@uv.mx (C.L.S.); rzenteno@uv.mx (R.Z.-C.); 2 Centro de Investigaciones Biomédicas, Universidad Veracruzana, Xalapa 91190, Mexico; 3 Facultad de Medicina, Universidad Veracruzana, Ciudad Mendoza 94740, Mexico; mvarela@uv.mx; 4 Centro de Alta Especialidad “Dr. Rafael Lucio”, Servicio Medicina Interna, Xalapa 91020, Mexico; trujirivera@hotmail.com; 5 Instituto de Biotecnología, Universidad Nacional Autónoma de México, Cuernavaca 62210, Mexico; espinosa@ibt.unam.mx (R.E.); mayret@ibt.unam.mx (M.G.); 6 Centro de Investigación Sobre Enfermedades Infecciosas, Instituto Nacional de Salud Pública, Cuernavaca 62100, Mexico; cramos@insp.mx; 7 Instituto Nacional de Medicina Genómica, Ciudad de México 14610, Mexico; jiperez@inmegen.gob.mx; 8 Facultad de Nutrición, Universidad Autónoma del Estado de Morelos, Cuernavaca 62350, Mexico; vanessa.lopezg@uaem.edu.mx; 9 Jurisdicción Sanitaria No. VII, Servicios de Salud, Orizaba 94300, Mexico; baruchblack@hotmail.com; 10 Instituto de Investigaciones Cerebrales, Universidad Veracruzana, Xalapa 91190, Mexico; frojas@uv.mx

**Keywords:** human coronavirus, COVID-19, antibodies, seroprotection, SARS-CoV-2

## Abstract

The causes of the broad spectrum of severity in COVID-19 are unknown. A protective effect through humoral immunity from previous infections by viruses of the SARS-CoV-2 family could explain a mild form of this disease. This study aimed to address whether the presence of antibodies against human seasonal coronaviruses (HCoVs) could prevent severe manifestations of COVID-19. A cross-sectional study was carried out in 165 participants. The presence of pre-existent antibodies against the seasonal HCoV-OC43, HCoV-HKU1, HCoV-229E, and HCoV-NL63 were detected. From all of the seasonal HCoVs studied, it was only found that being seropositive to HCoV-229E presented an association (*p* = 0.012) with developing mild clinical symptoms of COVID-19 or being asymptomatic. Multinomial regression analysis showed that being seropositive to HCoV-229E is associated with mild or moderate clinical symptoms for COVID-19. Statistical analysis also showed that being female is associated with being asymptomatic for SARS-CoV-2 infection or developing mild COVID-19. A subgroup analysis taking only seropositive to HCoV-229E revealed that females are more likely to develop asymptomatic SARS-CoV-2 infection (OR = 27.242, 95% CI 2.092–354.706, *p* = 0.012). Our results suggest that previous infections by HCoV-229E could prevent more serious clinical manifestations of COVID-19, but these are not the only variables that influence this event.

## 1. Introduction

The severe acute respiratory syndrome coronavirus 2 (SARS-CoV-2) is of recent circulation in humans and causes the disease called COVID-19 (Coronavirus disease 2019). The biological characteristics of SARS-CoV-2 allowed for its rapid spread, in such a way that, 17 months after its outbreak, it caused more than 184 million cases and 4 million deaths, with a global lethality rate of 2.1% [1]. SARS-CoV-2 is a coronavirus (CoV) with a ribonucleic acid (RNA) genome of positive polarity that belongs to the *Coronaviridae* family, which is divided into four genera: α, β, γ, and δ. The seasonal CoVs strains that infect humans (HCoVs) belong to the genera α (HCoV-NL63 and HCoV-229E) and β (HCoV-HKU1 and HCoV-OC43), and are associated with frequent and seasonal acute upper respiratory infections [2]. Emerging CoVs, such as the severe acute respiratory syndrome virus (SARS-CoV) and the middle east respiratory syndrome virus (MERS-CoV) that appeared in 2003 and 2012, respectively, are the cause of a severe respiratory infection with a high mortality [3].

It is estimated that about 40% to 45% of infected people with SARS-CoV-2 are asymptomatic [4]. In people who develop COVID-19, the clinical manifestations can be from mild to severe and even death [5,6]. The factors linked to the severity of COVID-19 are unknown with certainty; however, advanced age, gender, diabetes, hypertension, obesity, immunodeficiencies, and an exacerbated immune response are related to more severe clinical conditions [7,8,9]. 

One of the topics studied around SARS-CoV-2 infection is the role of humoral immunity induced by previous HCoVs infections in the development of COVID-19 disease. A recent study (preprint), reported that the presence of antibodies against HCoVs in the sera collected before the onset of the COVID-19 (pre-COVID-19) did not block the entry of the virus in a neutralization test with SARS-CoV-2 [10]. However, a retrospective epidemiological study in hospitalized patients found that recent HCoVs infections up to 440 days prior to contracting SARS-CoV-2 are associated with a lower admission to the intensive care unit, lower mortality, and higher survival [11]. 

The risk factors related to the broad spectrum of clinical manifestations reported in SARS-CoV-2 infection are still unknown. Given that previous infection by HCoVs could explain the different degrees of severity of COVID-19, in this study, it was investigated whether the presence of antibodies against seasonal HCoVs could confer a protective effect against developing a severe clinical form of COVID-19.

## 2. Materials and Methods

### 2.1. Study Design, Subjects, and Samples Collection 

A cross-sectional exploratory study was performed. The inclusion criteria were to be positive for SARS-CoV-2 infection by qRT-PCR for COVID-19 participants or by serology for 24 asymptomatic participants, 11 mild cases, and 1 moderate case. The serological test used was validated by the Mexican authorities (COFEPRIS) for the serological diagnosis of SARS-CoV-2 infection. The patients had at least 27 days after the onset of the symptoms and were all older than 18 years old. Blood samples were taken from 165 participants during August and September 2020 after signing an informed consent. The BD Vacutainer^®^ collection tubes with a coagulation activator were used. The samples were centrifugated at 3500 rpm for 5 min and the sera were stored at −80 °C until processing. 

### 2.2. Collection of Clinical Information and Severity Classification

The clinical and sociodemographic variables of interest were collected through a questionnaire prepared by the doctors. The information was collected through face-to-face interviews with the patients before taking the sample and through reviewing the clinical records.

COVID-19 severity classification was performed on the eight-category ordinal scale of the National Institutes of Health (NIH) of the United States of America [12,13].These categories were extrapolated to four groups based on the following criteria:

Asymptomatic: includes category one, which corresponds to non-hospitalized patients without a limitation of activities. They had no signs or symptoms of COVID-19.

Mild: includes non-hospitalized patients with physical activity limitations.

Moderate: includes hospitalized patients with oxygen requirements through a nasal catheter or reservoir mask.

Severe: includes hospitalized patients with oxygen requirements due to non-invasive mechanical ventilation (which may be due to high flow oxygen therapy) or invasive mechanical ventilation (which may be due to extracorporeal membrane oxygenation ECMO). 

All of the hospitalized patients required oxygen in this study. The possible death of participants during the development of the study is not ruled out.

### 2.3. ELISA for The Detection of IgG of Seasonal HCoVs

The ELISA technique for each HCoV was adapted from a previously established protocol [14]. Briefly, 96-well plates for immunoassays (#3590; Corning, Union City, CA, USA) were sensitized overnight at 4 °C with 50 μL per well of the respective recombinant protein for each HCoV (at a concentration of 2 μg/mL). Afterward, the plates were washed once with PBS and 200 μL per well of 1% albumin (BSA) in PBS was added as a blocking solution for 1 h at 37 °C. Then the blocking solution was removed and 50 μL of the sera not diluted were added in duplicate for 1 h a 37 °C. After this time, the plates were washed three times with a solution of PBS with 0.1% tween 20 (PBST 0.1%) and 50 μL per well were added of a 1:500 dilution of mouse anti-human IgG-alkaline phosphatase (#05-4222; Invitrogen, Carlsbad, CA, USA) in PBST 0.1% with 0.01% albumin for 1 h at 37 °C. Finally, the plates were washed again with PBST 0.1% and 50 μL per well of alkaline phosphatase substrate (#S0942; Sigma Aldrich, St. Louis, MO, USA) were added. Plates were incubated at room temperature and protected from the light until the appearance of color, approximately 15–45 min depending on the HCoV. The plates were read at 405 nm.

Sera from patients not reactive to HCoVs were used as a negative control. Cut-off points were established for each HCoV by calculating the standard deviation between the highest and the lowest optical density (O.D.), three standard deviations were added to the highest value as reported [15]. The O.D. cut-off values were 0.366 for HCoV-NL63 and HCoV-229E, 0.336 for HCoV-HKU1, and 0.291 for HCoV-OC43.

The recombinant proteins used in the ELISA technique were: 1 HCoV-NL63 S1 domain (#40600-V08H; Sino Biological, Wayne, PA, USA), HCoV-OC43 S1 + S2 domain (#40607-V08B; Sino Biological), HCoV-HKU1 S1 domain (#40021-V08H; Sino Biological), and HCoV-229E S1 domain (#400601-V08H; Sino Biological). Proteins were reconstituted and stored according to the manufacturer’s instructions. 

### 2.4. Statistical Analysis

The comparison of categorical variables, including the general characteristics of the participant such as age and gender, comorbidities, and the results for the α and β HCoVs were analyzed using the Chi-Square test; when the frequencies were less than five, Fisher exact test was used. Because the numerical variables did not comply with the assumption of normality, the median was used instead of the mean in their description. The Kruskal−Wallis test was applied to compare the median concentrations of the O.D. for each α and β HCoVs in each severity group. Spearman correlation was used to test if there was a correlation between the antibody titer for each of the α and β HCoVs and the protection of severity. 

The multinomial logistic regression model was used to estimate the variations in the level of severity with respect to the positive response for each HCoV. The model assumes independence between the options of the dependent variables. The dependent variable was “level of severity”, with the following four categories: asymptomatic, mild, moderate, and severe. Seasonal HCoVs, gender, and age group were considered as independent variables. The risk categories for each HCoVs were being seronegative, male, and over 42 years old. The results of the multinomial logistic regression were reported as an odds ratio (OR) with their corresponding 95% confidence intervals (95% CI) for all of the independent variables according to the category of the dependent variable, except for severe, which was considered as a reference category. As a first step, all variables were included in the model, and then manual backward elimination was used to eliminate the variables that were not associated with the level of severity. A level of 5% (α = 0.05) was used to retain the variables associated with the level of severity. The multinomial logistic regression model was also used for a subgroup analysis considering only those who were positive for HCoV-229E. The severe level was considered as a dependent variable. The statistical analysis was performed using the SPSS statistical package (IBM SPSS Statistics 21.0; SPSS Inc., Chicago, IL, USA).

## 3. Results

### 3.1. Study Population

The study included 165 participants, 49.7% (n = 82) were women and 50.3% (n = 83) were men. The 59.1% of the men presented moderate and severe symptoms (*p* ≤ 0.0001). The general median age without distinction between groups was 42 years old, so the participants were grouped into those under and over 42 years old. More than 70% of the participants under 42 years old were in the asymptomatic and mild groups (*p* = 0.045). Regarding comorbidities, diabetes was associated with a moderate condition of COVID-19 (*p* = 0.012) (Table 1).

Seropositivity to HCoV-229E and being female prevented the development of severe clinical manifestations of COVID-19.

The sera were analyzed using the ELISA technique to determine the presence of IgG antibodies against each of the seasonal HCoVs. Of the total population, 48% (80/165) were positive to HCoV-NL63 and 36% (60/165) to HCoV-229E, while 25% (41/165) were positive to HCoV-HKU1 and HCoV-OC43, respectively. A Chi-square test was applied to determine if there was an association between the degree of severity of COVID-19 and the presence of antibodies to HCoVs. It was observed that being positive to HCoV-229E is associated with presenting a mild condition of COVID-19 (asymptomatic or mild) (*p* = 0.012). No association was found with the other HCoVs (Table 2). 

The multinomial logistic regression model showed an association between being HCoV-229E seropositive and the level of COVID-19 severity; having antibodies to HCoV-229E made it more likely to have a mild (OR = 8.641, 95% CI 2.011–37.135, *p* = 0.004) or moderate clinical form of COVID-19 (OR = 4.754, 95% CI 1.135–19.914, *p* = 0.033). Women were more likely to have asymptomatic (OR = 11.285, 95% CI 2.212-57.569, *p* = 0.004) or mild COVID-19 (OR = 9.792, 95% CI 2.250−42.611, *p* = 0.002) (Table 3). 

A multinomial logistic regression analysis by subgroup, considering only those positive to HCoV-229E, was performed to know which sociodemographic variables influenced the level of severity. It was found that the model was consistent with the results of the bivariate analysis in showing an association between being seropositive to HCoV-229E, COVID-19 severity, and gender. The results show that being a female HCoV-229E seropositive made it more likely to have asymptomatic SARS-CoV-2 infection (OR = 27.242, 95% CI 2.092–354.706, *p* = 0.0012) compared with mild (OR = 4.611, 95% CI 0.738–28.815, *p* = 0.102) or moderate SARS-CoV-2 infection (OR = 0.789, 95% CI 0.111–5.606, *p* = 0.566). In the model, age was not associated with mild (OR = 4.611, 95% CI 0.738–28.815, *p* = 0.102), moderate (OR = 0.813, 95% CI 0.060–3.945, *p* = 0.499), or asymptomatic groups of patients (OR = 4.132, 95% CI 0.526–42.464, *p* = 0.177).

### 3.2. HCoV-229E Antibody Titer Is Related to The Severity Level of COVID-19

**Figure 1:**
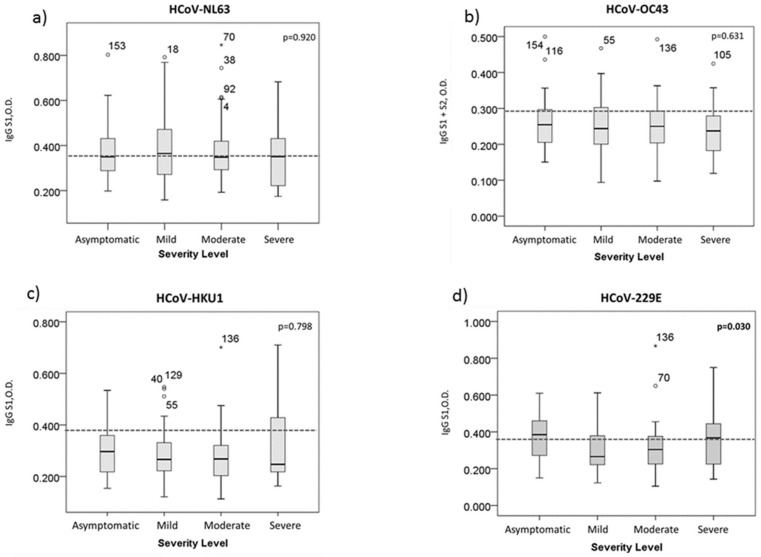
Boxplot of the optical densities (O.D.) of IgG antibodies for each of the HCoVs. (**a**) HCoV-NL63, (**b**) HCoV-OC43, (**c**) HCoV-HKU1, and (**d**) HCoV-229E. The dashed line indicates cut-off for each HCoV. **◦** Outliers: cases with the values between 1.5 and 3 box-lengths from the 75th percentile or 25th percentile. * The observation was attended. Extreme values cases with the values more than 3 box-lengths from the 75th percentile or 25th percentile.

The O.D. values obtained using the ELISA technique are a measure of the level of antibodies present in the serum samples from patients [16]. In this study, it was found that there was a difference in the antibody titers with HCoV-229E between the four groups of patients, namely: asymptomatic, mild, moderate, and severe (*p* < 0.030) (Figure 1). The distributions of the O.D. in the asymptomatic group were similar to the severe group. However, using Spearman’s correlation, a negative correlation (−0.081) was found between the antibody levels and severity, which, although weak, suggests that the higher the O.D., the less severity the condition of COVID-19 develops.

## 4. Discussion

SARS-CoV-2 is a novel virus in circulation that has caused a global health problem. Furthermore, it has generated an economic burden in trying to reduce its spread [17]. SARS-CoV-2 infection can be asymptomatic or generate clinical manifestations of variable severity. Different investigations have been developed to understand the pathology caused by SARS-CoV-2 and the factors that influence it. The cross-reactivity and protective effect of previous infections by seasonal HCoVs through humoral immunity could explain the mild forms of COVID-19 [18]. An unpublished study (preprint) reports that preexisting antibodies to HCoVs did not confer protection from acquiring SARS-CoV-2 infection or avoid severity in the study group, which was characterized by people who presented with a severe clinical condition of COVID-19 [10]. Interestingly, another study reported that hospitalized patients with previous infections by any seasonal HCoV were related to reducing clinical parameters associated with the severity of COVID-19, such as avoiding admission to intensive care or even death [11]. However, in that study, it was not found which of the seasonal HCoVs confered seroprotection [11]. Moreover, they associated being positive to any HCoVs in a range of 69 to 440 days prior to SARS-CoV-2 infection as the factor responsible for avoiding intensive therapy or death [11]. HCoVs infection was confirmed by RT-PCR [11]; it is unknown if all of the patients who developed COVID-19 had antibodies present for any of the HCoVs at or near to the time of SARS-CoV-2 infection. This may have played a role in the course of the disease, as it has been reported that mild infections do not induce seroconversion or that the humoral response generated is weak [19,20]. In this work, an analysis of seropositivity was carried out for each of the seasonal HCoVs strains related to COVID-19 severity. Our study is the first to establish an epidemiological association between the presence of preexisting antibodies to HCoV-229E and preventing a severe form of COVID-19. The bivariate analysis showed a higher probability of being asymptomatic or having a mild COVID-19 if a person was seropositive to HCoV-229E. The multivariate analysis also suggests a lower probability of having severe COVID-19 in seropositive people. This last finding is similar to the one that had already been reported for any seasonal HCoV [11].

Different studies have found that pre-COVID-19 sera show cross-reactivity against the complete S protein, the S2 domain, and the N protein of coronavirus [10,21]. None of them report reactivity against the S1 region or the receptor-binding domain (RBD), so the behavior of the pre-COVID-19 sera against the S protein could be due to cross-reactivity with the S2 region, in which shared epitopes have been found between SARS-CoV-2 and the known human coronavirus SARS-CoV, MERS-CoV, HCoV-HKU1, HCoV-OC43, HCoV-NL63, and HCoV-229E [22]. In this work, the S1 region (except for HCoV-OC43) was used to determine seropositivity. However, the mechanism that we proposed to explain the epidemiological association that we found, in part, is that preexisting antibodies against the S2 region, in some way, could be involved in seroprotection, as reported elsewhere [21,22]. The role of other antibodies against other viral proteins such as N is not ruled out. The molecular mechanism that supports the epidemiological evidence presented in this work has yet to be demonstrated. 

The presence of preexisting antibodies against HCoVs might not be the only reason that could explain the differences observed in the variations of the severity of COVID-19. Men have been reported to develop more severe clinical manifestations, as well as a higher mortality [23]. The distribution of men and women was similar in our study population. Despite this, the men presented a greater severity of COVID-19. The proportion of women with a mild presentation of the disease and who also had antibodies against HCoV-229E was higher compared with men. In addition, in this work, it was found that the probability of being an asymptomatic carrier of SARS-CoV-2 was higher in women, so there must be additional factors that, added to the presence of antibodies to HCoV-229E, provide a protective effect against the severity of the disease, which must be investigated in greater detail.

Based on the precedent that the clinical manifestations of a certain disease are related to the level of antibodies directed against the pathogen that induces such a humoral response [24,25] and, in turn, depends on the O.D., we consider the possibility that the antibody titers for HCoV-229E were related to the severity of COVID-19. We found that the O.D. is inversely related to severity; however, the correlation was very weak—almost inexistent. This suggests that not only the presence of antibodies against HCoV-229E has a positive impact on reducing the clinical manifestations of COVID-19. This is consistent with the fact that we found that other variables, such as gender, could influence the evolution of SARS-CoV-2 infection. 

One of the limitations of our study is that the serum samples were obtained in a range of 27 to 159 days after the date of the onset of symptoms or probable contact in the case of asymptomatic patients. The possibility that HCoV infection may have occurred during this period, that is, after the SARS-CoV-2 infection, is not ruled out, so we are developing a molecular analysis to verify our findings. Another limitation of this work is the sample size, which, due to the segmentation for the statistical analysis, generated small groups. A larger population would allow for more homogeneous groups.

## 5. Conclusions

This study is the first to perform an analysis according to the group of severity of SARS-CoV-2 infection, including asymptomatic patients and the other manifestations of COVID-19 (mild, moderate, and severe forms), and to relate it to an analysis of seropositivity to HCoVs. At the same time, it also proposes the individual association for each of the HCoVs, pointing to the previous infection by HCoV-229E as a possible protective effect against severe clinical manifestations of COVID-19. This protective effect could be higher if the person is a female. In this work, we studied the possible role that antibody-mediated immunity against HCoVs could have during SARS-CoV-2 infection, where molecular verification must be carried out. However, the role that innate and cellular immunity [26] could play during COVID-19 disease development is not ruled out [27].

## Figures and Tables

**Table 1:** General and clinical characteristics of the study population.

Characteristic	Classification of Severity					
	Asymptomatic	Mild	Moderate	Severe	Total	*p* Value
	n = 26	n = 74	n = 47	n = 18	n = 165	
**General characteristics**						
Patient gender						**<0.001**
Female	19 (23.2)	47 (57.3)	13 (15.9)	3 (3.7)	82 (100)	
Male	7 (8.4)	27 (32.5)	34 (41)	15 (18.1)	83 (100)	
Age						**0.045**
42 years old and under	12 (14.8)	45 (55.6)	17 (21)	7 (8.6)	81 (100)	
More than 42 years old	14 (16.7)	29 (34.5)	30 (35.7)	11 (13.1)	84 (100)	
**Comorbidities**						
Diabetes	2 (9.5)	7 (33.3)	12 (57.1)	0 (0)	21 (100)	**0.012**
Cardiovascular problems	3 (9.4)	10 (31.3)	15 (46.9)	4 (12.5)	32 (100)	0.058
Allergic diseases	2 (33.3)	3 (50)	1 (16.7)	0 (0)	6 (100)	0.531
Neoplasic lesions	0 (0)	1 (100)	0 (0)	0 (0)	1 (100)	0.747
Autoimmune diseases	1 (100)	0 (0)	0 (0)	0 (0)	1 (100)	0.257
Smoking	0 (0)	0 (0)	1 (100)	0 (0)	1 (100)	0.461

*p* values < 0.05 are in bold. Data are represented as n (%). Chi-square test was performed.

**Table 2:** Association of HCoV with the severity of SARS-CoV-2 infection.

Positive Results	Classification of Severity					
	Asymptomatic	Mild	Moderate	Severe	Total	*p* Value
	n = 26	n = 74	n = 47	n = 18	n = 165	
HCoV-NL63	12 (15.0)	38 (47.5)	22 (27.5)	8 (100)	80 (100)	0.925
HCoV-229E	15 (25.0)	21 (35.0)	14 (23.0)	10 (17.0)	60 (100)	**0.012**
HCoV-HKU1	11 (26.8)	17 (41.5)	8 (19.5)	5 (12.2)	41 (100)	0.111
HCoV-OC43	7 (17.1)	19 (46.3)	13 (31.7)	2 (4.9)	41 (100)	0.551
α y β HCoV	2 (16.7)	6 (50.0)	3 (25.0)	1 (8.3)	12 (100)	0.975
αHCoV	8 (20.0)	15 (37.5)	11 (27.5)	6 (15.0)	40 (100)	0.559
βHCoV	5 (29.4)	7 (41.2)	4 (23.5)	1 (5.9)	17 (100)	0.407

*p* values < 0.05 are in bold. Data are represented as n (%). Chi-square test was performed and with frequencies less than five, Fisher exact test was used. HCoV—human coronavirus.

**Table 3:** Multinomial regression model for positive HCoV, gender, and age associated with COVID-19 severity.

Seasonal	Classification of Severity											
	Asymptomatic				Mild				Moderate			
HCoV	OR	CI 95%		*p*	OR	CI 95%		*p*	OR	CI 95%		*p*
		LL	UL	value		LL	UL	value		LL	UL	value
Female	**11.285**	**2.212**	**57.569**	**0.004**	**9.792**	**2.250**	**42.611**	**0.002**	2.079	0.455	9.504	0.345
>42 years old	1.459	0.322	6.623	0.624	3.052	0.789	11.807	0.106	0.781	0.199	3.068	0.724
HCoV-NL63	0.818	0.163	4.107	0.807	0.342	0.081	1.450	0.146	0.488	0.114	2.093	0.334
HCoV-229E	1.336	0.264	6.757	0.726	**8.641**	**2.011**	**37.135**	**0.004**	**4.754**	**1.135**	**19.914**	**0.033**
HCoV-HKU1	0.497	0.091	2.709	0.419	0.569	0.116	2.796	0.488	2.321	0.415	12.992	0.338
HCoV-OC43	0.147	0.014	1.554	0.111	0.138	0.015	1.305	0.084	0.163	0.017	1.548	0.114

*p* values < 0.05 are in bold. HCoV—human coronavirus; OR—odds ratio; LL—lower limit; UL—upper limit; CI 95%—confidence interval of 95%.

## Data Availability

The data underlying this article will be shared upon reasonable request to the corresponding author.
